# Prevalence of PTSD and other mental disorders in UK service personnel by time since end of deployment: a meta-analysis

**DOI:** 10.1186/s12888-016-1038-8

**Published:** 2016-09-22

**Authors:** Roberto J. Rona, Howard Burdett, Samantha Bull, Margaret Jones, Norman Jones, Neil Greenberg, Simon Wessely, Nicola T. Fear

**Affiliations:** 1 King’s Centre for Military Health Research, King’s College London, Weston Education Centre, 10 Cutcombe Rd, London, SE5 9RJ UK; 2 University of Manchester, Section of Clinical & Health Psychology, Manchester, UK; 3 Academic Department of Military Mental Health, King’s College London, London, UK

**Keywords:** Alcohol misuse, Impact of deployment over time, Posttraumatic stress disorder (PTSD), Psychological distress, Prevalence trends

## Abstract

**Background:**

US studies have shown an increase of posttraumatic stress disorder (PTSD) and depression, but not alcohol misuse related to time of assessment since returning from deployment. We assessed if similar trends occur in the UK Armed Forces.

**Methods:**

We selected UK studies based on our data base of King’s Centre for Military Health Research publications from 2006 until January 2016 with at least one of the following measures: PTSD checklist-civilian version (PCL-C), the General Health Questionnaire (GHQ-12) and the Alcohol Use Disorders Identification Test (AUDIT). The studies included personnel assessed for these outcomes after their most recent deployment. A search in Medline, Psycho-Info and Embase confirmed that no relevant publication was missed.

**Results:**

Twenty one thousand, seven hundred and forty-six deployed personnel from nine studies contributed to the meta-analyses by time since end of deployment in the PTSD analysis. The number of studies for period of time varied from two to four studies. The trend by time-category of questionnaire completion since returning from deployment were for PTSD β = 0.0021 (95 % CI −0.00046 to 0.0049, *p* = 0.12), for psychological distress β = 0.0123 (95 % CI 0.005 to 0.019, *p* = 0.002) and for alcohol misuse β = 0.0013 (−0.0079 to 0.0105, *p* = 0.77).

**Conclusions:**

There was no evidence that the prevalence of PTSD and alcohol misuse changed according to time since the end of deployment over a three-year period, but there was evidence for an association with increasing psychological distress.

**Electronic supplementary material:**

The online version of this article (doi:10.1186/s12888-016-1038-8) contains supplementary material, which is available to authorized users.

## Background

There is considerable heterogeneity in the prevalence of posttraumatic stress disorder (PTSD) between international military studies [1]. Some studies have shown that the prevalence of PTSD increases with time of assessment since the end of last tour of duty [2, 3] while UK studies do not show an increase [1] or show a slight increase [4]. Some of the heterogeneity in prevalence rates over time may be due to differences between settings related to nations, as policies about deployment lengths, for example, tend to differ between nations. If some of the heterogeneity due to the diverging policies between nations can be removed differences between studies in the relationship by time of assessment since end of deployment and the prevalence of mental disorders could be understood better.

Individuals change their mental health status overtime; it has been shown that delayed presentation may be found in as many as 70 % of PTSD cases over a three year-period [5]. Twenty percent of cases of delayed PTSD are seen in personnel who previously showed some symptoms and later on became full cases [5, 6]. At the same time, we found that 66 % of individuals who reported symptoms consistent with probable PTSD at the baseline assessment remitted or partially remitted at follow-up, on average three years later [7]. The issue of interest to assess is whether the prevalence of mental disorders varies according to time of assessment. This is important because it may be a consideration for preventive interventions such as screening for mental disorders in the military in which it may be construed that assessment of personnel would be potentially more beneficial when the prevalence of mental disorders are higher or have stabilised.

We have carried out several studies based on different samples of UK personnel, which used similar measures for assessment of mental ill health. These include studies during deployment [8, 9], immediately after deployment [10, 11], three to four months post deployment and two cohort studies started in 2001 and 2003 [4, 12, 13]. These studies included personnel deployed to operations in Iraq and Afghanistan. The aim of this study is to assess the prevalence rates of PTSD, psychological distress and alcohol misuse at the time of questionnaire completion in relation to last deployment. This study eliminates the effects of international differences such as deployment length and time between deployments between studies because they are based on the UK military, although the aims between studies may have been different.

## Methods

### Study selection

The studies selected were those carried out by our group, the King’s Centre for Military Health Research (KCMHR) and Academic Centre for Defence Mental Health (ACDMH), from 2006 until January 2016 in relation to the Iraq and Afghanistan conflicts. We have based the study in KCMHR and ACDMH because after carrying a search in Medline, Psycho-Info and Embase we would not have added any contributions relevant to our study (see Additional file 1). Furthermore, the database included all our papers, while the literature search would have missed some of our studies. Altogether these two collaborative groups working in the same department have published 320 papers during the period, 75 original studies dealt with deployed personnel to Iraq and/or Afghanistan. Nine papers were considered the source of the datasets of six projects carried out by KCMHR and ACDMH that contributed to this analysis, or guided us to independent datasets which should be included in the analysis [11, 14], but 66 reports were duplication using material from the same nine papers. Table 1 shows the details of the nine datasets in the analysis that are based on six projects: three datasets from the main cohort project [4, 13, 15], two datasets from the Operational Mental Health Needs Evaluation (OMHNE) project [8, 16]; one dataset was a cluster randomized controlled trial (cRCT) project [10] and the POST project (also a cRCT) contributed with one data set [14], another dataset from a screening project carried out in 2001 but followed up at the same time as the main cohort project [12], and a data set from a cross-sectional project [11]. In summary four projects contributed with one dataset to the analysis, the OMHNE project contributed with two datasets and the main cohort project with three datasets. For ease of expression we will talk of nine studies in the analysis from now on. The studies included at least one of the following measures: General Health Questionanire-12 (GHQ-12) [17], PTSD check list civilian version (PCL-C) [18] or Alcohol Use Disorders Identification Test (AUDIT) [19]. The cut-off used to determine prevalence was a score of 50 or more for the PCL-C (range 17 to 85), a score of 4 or more for GHQ-12 (range 0 to 12), and score of 16 or over for the AUDIT (range 0 to 40). These are cut-offs used in most of those studies. Table 1 shows the details of the selected studies. Some of the studies collected data at precise periods [8–11, 14], while in other studies questionnaires were returned over a post deployment period between less than 3 months and up to 3 years [4, 12, 13, 15]. These studies included a non-deployed group which was excluded from these analyses. In those studies in which completion occurred over a long period the sample was divided into the following categories in relation to the end of deployment: less than 3 months, 3–5.9 months, 6–11.9 months, 12–17.9 months, 18–23.9 months and 24 or more months.

**Table 1:** Characteristics of the KCMHR and ADDMH UK Armed Forces studies

Deployment period	Study	Study design	Sample size	Method of data collection	Period of data collection	Stage in deployment cycle	Deployment location	Outcomes^a^ studied
Mid-deployment	Mulligan et al. [8]	Purposive sampling^b^	611	Self-report questionnaire	Jan–Feb 2009	Mid	Iraq	GHQ-12 PCL-C
	Jones et al. [9]	Cluster-based purposive sampling	2794	Self-report questionnaire	Winter 2010/Jul–Aug 2011	Mid	Afghanistan	GHQ-12 PCL-C
Returning from deployment	Mulligan et al. [10]	Cluster RCT	2443	Self-report questionnaire	Mar–Apr 2009	Decompression	Afghanistan	PCL-C GHQ-12
	Banwell et al. [11]	Cross-sectional	2580	Self-report questionnaire	March–Apr/ Sept–Oct 2011	Decompression	Afghanistan	PCL-C GHQ-12
Post-deployment	Burdett et al. [14]	Cluster RCT	8719	Computer-based self-report questionnaire	Oct 2011–Feb 2013	6–12 weeks post-deployment	Afghanistan	PCL-C AUDIT
	Fear et al. [15]	Cohort study	3578	Self-report questionnaire, postal or visit	Jun 2004–Mar 2006	<6 months post-deployment, 6–11 months, 12–17 months, 18–23 months, 24+ months	Iraq	AUDIT
	Hotopf et al. [13]	Cohort study	4722		Jun 2004–Mar 2006		Iraq	GHQ12 PCL-C
	Rona et al. [12]	Cohort study	669		Jun 2004–Mar 2006		Iraq	PCL-C GHQ-12 AUDIT
	Fear et al. [4]	Cohort study	6715		Nov 2007–Sep 2009		Iraq and Afghanistan	PCL-C GHQ-12 AUDIT

^a^ *GHQ-12* 12-item General Health Questionnaire, *PCL-C* Post-traumatic stress Check List – Civilian version, *AUDIT* 10-item WHO Alcohol Use Disorders Identification Test. AUDIT-3

^b^ Selecting a diversity of locations to represent all levels of combat exposure and recruiting those available in the bases in each pre-selected locations

Altogether we identified nine suitable studies from six datasets. Eight of the studies were independent from each other, but two studies have a percentage of individuals who took part in the two phases of the study. [4, 13] However, these two studies are not duplicate studies, as questionnaire completion were carried out at two different occasions from 2004 to 2006 (Phase 1) and 2007 to 2009 (Phase 2). The time of completion for the individuals participating more than once had a mild correlation of 0.17. One of the published papers was based in a subsample of the full study so the data extraction for this study was obtained from the Army and Royal Marines full data set rather than the published paper [11].

This piece of research adhered to PRISMA guidelines/methodology.

### Analysis

We extracted the following variables from each study: number of participants, prevalence of the outcome of interest including the 95 % confidence interval (CI). Using this extracted data we carried out a meta-analysis of prevalence for each period of assessment when more than one study was available for the period. Forest plots for each outcome (PCL, GHQ-12 and AUDIT) and period of questionnaire completion in relation to deployment were carried out using a random effect analysis (available from the authors), but only the overall assessment is given in this paper together with the degree of heterogeneity of the weighted assessment. Heterogeneity for each forest plot was estimated with I^2^, a measure that assesses heterogeneity as opposed to variation attributed to chance. I^2^ ranges between 0 and 100 %. Linear and squared trend analyses were carried out for each condition separately and omitting the mid-deployment group in this estimate using STATA command metareg [prevalence variable] time, wsse[standard error]. The analysis was carried out using STATA 11.2 (Stata Corporation, USA). The mid-deployment group was omitted because the main aim of the study was the association between prevalence and time since the end of deployment, but we included for completeness the mid-deployment in the results.

## Results

Data from 23,037 deployed personnel contributed to the PTSD analysis. The size of the studies ranged from 611 to 8719 individuals (Table 1). Most studies included all the three services (Naval Services, Army, Royal Air Force), except the post deployment screening for mental illness study (POST) that included only the Army and Royal Marines deployed to Afghanistan and for this analysis the data extracted from deployment samples [11]. Five studies were based on a representative sample of the UK Armed Forces, two mid-deployment studies aimed to obtain a purposive sample (participants included from a wide range of locations within the theatre of operations, but researchers did not have a register of those in each location beforehand), and two cRCT (which aimed to include all those in a given platoon or company formation) and one of the studies that sampled personnel just returning from deployment at military bases in Cyprus [11]. All studies used a self-administered questionnaire, except the POST study which used a computer based self-reported questionnaire with a two phase assessment (abridged questionnaire and, if positive, the complete questionnaire) [14]. Service-demographic information is given in Table 2 for eight studies, as two of the studies have the same characteristics, except the outcome assessed [13, 15]. There were differences between the eight studies in the percentage of participating females: most studies included between seven per cent and 11 % females, but two studies contained two per cent and three per cent, commissioned officers (CO) percentage varied from five percent to 23 %, and reserves from zero per cent to 14 %. The percentage of personnel under 30 years old varied from 40 to 73 % and the Army was, as expected, the largest component of all the studies, but varied from 53 to 86 %. Some of these variations were due to study design, as two of these studies over-sampled reserves [4, 13] or did not include reserves (the POST study and pre-deployment screening study), gender variation with period of assessment (women were less represented in the mid-deployment and POST studies), or selection by platoon formation including greater numbers of younger personnel.

**Table 2:** Demographic and service characteristics of the studied samples included in the analysis

References	Females N (%)	Commissioned officer N (%)	Reservists N (%)	Under 30 years N (%)	Army N (%)
Banwell et al. [11]	93 (8)	223 (17)	71 (6)	676 (54)	1213 (94)
Fear et al. [4]	651 (10)	1438 (21)	972 (14)	2659 (40)	4669 (70)
Hotopf et al. [13]^a^	432 (8)	814 (17)	786 (9)	1862 (42)	3066 (64)
Jones N et al. [9]	211 (8)	313 (11)	158 (6)	1901 (68)	2446 (88)
Mulligan et al. [8]	69 (11)	76 (12)	110 (4)	1781 (71)	497 (82)
Mulligan et al. [10]	41 (2)	229 (9)	108 (4)	1751 (71)	1332 (55)
Rona et al. [12]	44 (7)	155 (23)	0 (0)	167 (25)	352 (53)
Burdett et al. [14]	251 (3)	359 (5)	0 (0)	6317 (73)	7535 (86)

^a^Fear et al. [15] has the same demographic and service characteristics as Hotopf et al. 2006

**Fig. 1:**
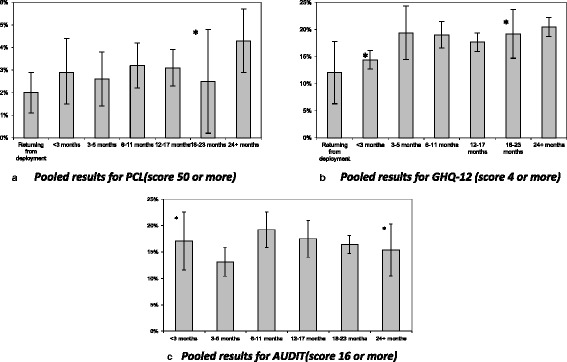
Trends in the prevalence of (**a**) probable PTSD, (**b**) psychological distress and (**c**) Alcohol misuse by time of questionnaire completion in relation to end of last deployment (excluding mid-deployment group). * Significant heterogeneity between studies in this time period

There were three studies contributing to all categories from 3 months post-deployment upwards, two studies for the mid-deployment period, two studies in which assessment took place when returning from deployment and four in which assessment was carried out between less than three months since the end of deployment. The smallest group included 863 subjects. The prevalence of PTSD was slightly lower in all groups up to less than 6 months (2.0 to 2.9 %) than those completing the questionnaire later on (between 2.5 and 4.3 %) (Table 3, Fig. 1a). The group completing the questionnaire between 18 and 23 months was significantly heterogeneous between studies (I^2^ = 92.4 %, *p* < 0.001). The trend between categories of prevalence of PTSD from just returning from deployment onward was non-significant (coefficient 0.0021, 95 % CI −0.0006 to 0.0049, *p* = 0.12). There was not significant association after including a squared term. The contribution of each study to a period is available from the authors.

**Table 3:** Pooled prevalances and heterogeneity between samples (meta-analysis performed with random effect model)

Time since end of deployment	Studies (total N)	PCL-C^1^		GHQ^1^		AUDIT^1^		
		% (95 % CI)	I^2^%, *p*-value	% (95 % CI)	I^2^%, *p*-value	Studies (total N)	% (95 % CI)	I^2^%, *p*-value
Mid-deployment	2 (3405)	2.5 (1.6–3.4)	59.2, 0.086	17.6 (15.2–20.0)	66.6, 0.05	-	-	-
Returning from deployment	2 (3712)	2.0 (1.1–2.9)	68.3, 0.08	12.1 (6.3–17.8)	96.4, <0.001	-	-	-
<3 months post-deployment	4 (9398)	2.9 (1.5–4.4)	58.6, 0.065	14.4 (12.7–16.1)	6.1, 0.29	4 (9253)	17.1 (11.6–22.6)	78.9, 0.003
3–6 months post-deployment	3 (863)	2.6 (1.4–3.8)	0.0, 0.39	19.4 (13.9–22.5)	49.7, 0.14	3 (734)	14.1 (10.4–15.9)	0.0, 0.70
6–11 months post-deployment	3 (1332)	3.2 (2.2–4.2)	0.0, 0.86	19.0 (16.6–21.5)	0.0, 0.52	3 (1072)	19.2 (15.8–22.6)	9.8, *p* = 0.33
12–17 months post-deployment	3 (2062)	3.1 (2.3–3.9)	0.0, 0.55	17.7 (15.9–19.4)	0.0, 0.40	3 (1819)	17.5 (14.0–21.0)	63.7, *p* = 0.064
18–23 months post-deployment	3 (2228)	2.5 (0.2–4.8)	92.4, < 0.001	19.2 (14.7–23.7)	76.4, 0.014	3 (2025)	16.4 (14.7–18.1)	0.0, *p* = 0.88
24+ months post-deployment	3 (2993)	4.3 (2.9–5.7)	54.5, 0.11	20.5 (18.7–22.2)	9.6, 0.33	3 (2880)	15.4 (10.5–20.3)	86.5, *p* = 0.001

*GHQ-12* 12-item General Health Questionnaire, *PCL-C* Post-traumatic stress Check List – Civilian version, *AUDIT* 10-item WHO Alcohol Use Disorders Identification Test

There was a slight increase in the pooled prevalence of psychological distress based on GHQ-12 in relation to the time of questionnaire completion and end of deployment (Table 3, Fig. 1b). Those completing the questionnaire 24 or more months after the end of deployment had the highest prevalence. There was a significant trend in the prevalence between categories of psychological distress and time of questionnaire completion since returning from deployment onward (0.0123 (95 % CI 0.005 to 0.019, *p* = 0.002). Heterogeneity between studies in the weighted prevalence assessment was found in those completing on returning from deployment (I^2^ = 96.4 %, *p* < 0.001) and those completing the questionnaire between 18 and 23 months post-deployment (I^2^ = 76.4 %, *p* = 0.014). The inclusion of a squared term to the analysis was non-significant. The contribution of each study to a period is available from the authors.

The pooled prevalence of scores of 16 or more on the AUDIT was highest in those completing the questionnaire between 6 and 11 months after the end of deployment and thereafter the prevalence started to decrease (Table 3, Fig. 1c). Those who participated in the mid-deployment study were not asked the AUDIT questions as alcohol consumption during deployment is prohibited. Heterogeneity between studies was found in those completing the questionnaire 24 or more months after the end of deployment. The trend between the prevalence of alcohol misuse and time of questionnaire completion from the end of deployment was not significant (β = 0.0013, 95 % CI −0.0079–0.0105, *p* = 0.77). The inclusion of a squared term in the model was not meaningful. The contribution of each study to a period is available from the authors.

## Discussion

We found no change in the prevalence of PTSD and alcohol misuse by time of questionnaire completion since end of deployment, although there was a slightly smaller prevalence of PTSD in the groups up to less than 6 months than those assessed later on. However, there was an association in the prevalence of psychological distress with time of assessment since the end of deployment.

In contrast to the results of this study, all the US studies have shown an increase in the prevalence of PTSD in relation to post-deployment time of questionnaire completion [2, 3, 20–23].

We have previously reported about the prevalence of PTSD according to time of questionnaire completion since deployment in the UK military [1]. In this study, we have extended those observations as follows: more outcomes (psychological distress and alcohol misuse), a longer time span of observation, more detailed assessment of time trends, increased the number of samples from two to eight and estimates are based on at least 863 observations in each time period category in the analysis.

### PTSD prevalence by time of questionnaire completion since end of deployment

A different approach has been taken to assess PTSD trajectories - latent class growth modelling.[24–28] These methods do not define classes at the outset, as they are selected according to goodness of fit and these classes are frequently inconsistent between studies. An increase in PTSD prevalence according to time since deployment seems to occur in a US study and a Danish study of this type [24, 29]. It is difficult to infer prevalence trends based on latent class growth modelling as the aim of these analyses are to characterize the heterogeneity in the evolution of PTSD over time, but it appears to be an increase because in the early post-deployment assessments the percentages compatible with the definition of PTSD were smaller than the percentage compatible with this threshold in the latest assessments.

It is plausible that prevalence of PTSD may be low soon after returning from deployment, as service personnel may be reluctant to provide any information that may jeopardize immediate post-deployment leave; and a sense of relief to be back home could play a part. It is also plausible that during deployment personnel are reluctant to demonstrate weakness by reporting mental ill health [30]. However, the prevalence of PTSD continues to be low for up to 5 months during the early post-deployment period when these reasons do not hold. Other factors may play a part in the prevalence rates including- types of enlistment (reserve and regulars) [4], role of participants (combat or other), [13] whether participants have left the armed forces, as it is more likely to be the case in those completing the questionnaire at a later time after last deployment [31], and the level of perceived support from relatives and friends by service personnel [32].

### Other outcomes prevalence by time since end of deployment

We found an increase in the prevalence of psychological distress based on the GHQ-12 with time since returning from deployment of psychological distress. The graphic trend is not consistent as the mid deployment group had a higher prevalence of psychological distress so the trend is mainly due to the low prevalence of psychological distress in those just returning from deployment and those less than 3 months since the end of deployment. We discuss this finding in the context of depression, although we have to be cautious as our measure is broader than measures of depression used in other studies. Depression has been found to increase by time since the end of deployment in US studies [2, 23, 33]. The prevalence rates increased from five per cent to ten per cent in active personnel and four per cent to 13 % in National Guard and Reserves using the PHQ-2 between the post-deployment health assessment and reassessment [2]. Thomas and colleagues found an increase between the third and 12th month’s post-deployment in National Guards personnel, but not in active duty personnel, using the PHQ-9 [23]. Veterans Affairs Health Care data for the period 2002–2008 showed an increase in diagnosis since deployment using International Coding of Diseases- 9 (ICD-9-Clinical Modifications) [33]; these results are difficult to interpret in the context of our analysis, as the denominator is based on consultations to Veteran Administrative Services which are different to population at risk denominators, and it is uncertain whether there were increases in first consultation within cohort. Another study showed an increment in physical symptoms of personnel over time in a clinical sample [34]. It is well known that measures of unexplained physical symptoms are associated with depression and PTSD [35]. In summary most of the literature seem to concur that there is an increase of depression or proxy measures and time since the end of deployment.

The differences between studies in relation to alcohol misuse and the end of deployment are of smaller magnitude than those related to PTSD and depression. Our findings were consistent with the results of a US study which showed no significant trend [23]. Another US study reported a small increase in diagnosed alcohol misuse disorders over time [33]. We have previously reported in a longitudinal analysis that there was an increase in alcohol consumption immediately after the end of deployment that decreased over time [36].

It is difficult to provide an explanation for the small increase of the prevalence of psychological distress in contrast to the unchanged prevalence for PTSD and alcohol misuse. We could speculate that when service personnel just returns from deployment they experience a sense of relieve and enjoyment to be close to friends and family and that sense of wellbeing might dwindle over time. In the case of PTSD the situation is more complex as some of those experiencing PTSD symptoms when just returning may improve over time while others that did not have symptoms just after returning home start to develop symptoms [5]. In the case of alcohol misuse most service personnel have a well-established pattern of behavior that remains unchanged over time (paper in preparation). An alternative explanation in the contrast between the unchanged prevalence of PTSD and increasing prevalence of psychological distress is that is easier to demonstrate a trend in a condition with higher prevalence than in a condition with lower prevalence, such as PTSD in the UK military.

### Strengths and weaknesses

The strengths of this study are the use of several large data sets with different samples and aims, but which used similar tools, settings and approaches to data collection. The groups by period were all large, at least 863 subjects. As usual in meta-analyses, the main weakness of this study based on collation of summary data is that adjustment for possible confounders such as enlistment type, rank, gender, ex-serving personnel, type of enlistment and combat role could not be carried out. An impediment for merging the data sets is that the studies do not use the same items in the questionnaire, for example the cRCTs were mainly restricted to the measures related to the aims of the studies. The slightly lower prevalence of PTSD and psychological distress could have been influenced by the lower percentages of women in the POST and those studies carried out soon after returning from deployment. However, those were the same studies that had a lower percentage of reserves that are at a higher risk of PTSD and depression in UK studies [4, 13]. In addition the POST study has the lowest percentage of commissioned officers which are a group known to report a lower prevalence of mental ill health in military studies [31]. These three characteristics operate in different directions and may well have altered the effects in relation to the weighted prevalence rates. Although we recommend caution in the interpretation of these results, it is worth pointing out that the prevalence rates between the studies in our analysis are usually similar.

Similarly, there are more personnel who left service in the groups completing the questionnaire at a later time since the end of deployment. This may have slightly increased the prevalence of PTSD and psychological distress most likely because those with a mental disorder tend to leave the forces earlier. The usual way to assess the impact of these covariates in a meta-analysis would have been to stratify for each of these characteristics separately, but the number of studies within each category was small (between two and four studies) and thus there were insufficient studies to carry out stratified analyses.

### Implications

There is little doubt that the trends of PTSD since the end of deployment are very different between the UK and US Armed Forces. The UK prevalence rates do not vary or vary minimally by time of questionnaire completion since end of deployment. It is possible that the differences between the results in the UK and US military could be explained by differences in deployment experiences. We believe that this is unlikely as the UK and US military fought the same conflicts, facing the same tactics, and since 2005 having similar fatality rates [37]. Although we suspect that differences in compensation and health care policies, social support, deployment length and, possibly, time between deployments may be operating to explain the difference between the US and UK armed forces, we still have limited knowledge of the reasons for these striking differences. The contrasting results are less of an issue in relation to the other outcomes in the study psychological distress based on GHQ-12 that can be considered a proxy measure of depression. Likewise the results for alcohol misuse are more consistent between the two countries. Further progress could be made to understand changes in prevalence over time by assessing the trajectories of PTSD, psychological distress and alcohol misuse over time [24, 25, 28]. Such studies would allow us to learn about the evolution of these outcomes within individuals. As personnel with a combat role experience a higher prevalence of PTSD than those with other roles, an analysis restricted to those with a combat role might be helpful to assess any departure from the results reported in this paper.

## Conclusions

Time of questionnaire completion in relation to end of deployment does not have a strong impact on the prevalence of PTSD in the UK military, in contrast to results in the US military, but the trend in relation to other outcomes are more consistent between the two countries.

## Additional file

Additional file 1: Search strategy of UK studies that could be potentially relevant for inclusion in this study. (DOCX 195 kb)

## Abbreviations

ACDMH: Academic Centre for Defence Mental Health
AUDIT: 10-item WHO Alcohol Use Disorders Identification Test
CI: Confidence interval
CO: Commissioned officer
cRCT: Cluster Randomised Controlled Trial
GHQ-12: 12-item General Health Questionnaire
ICD-9: International Coding of Diseases- 9
KCMHR: King’s Centre for Military Health Research
PCL-C: Post-traumatic stress Check List – Civilian version
POST: Post Operational Screening Trial
UK: United Kingdom
US: United States
