# Supplementary figures and images for: Prevalence of PTSD and other mental disorders in UK service personnel by time since end of deployment: a meta-analysis
Source: BMC Psychiatry. 2016 Sep 22;16:333. doi: 10.1186/s12888-016-1038-8 (PMC5034433; doi:10.1186/s12888-016-1038-8)

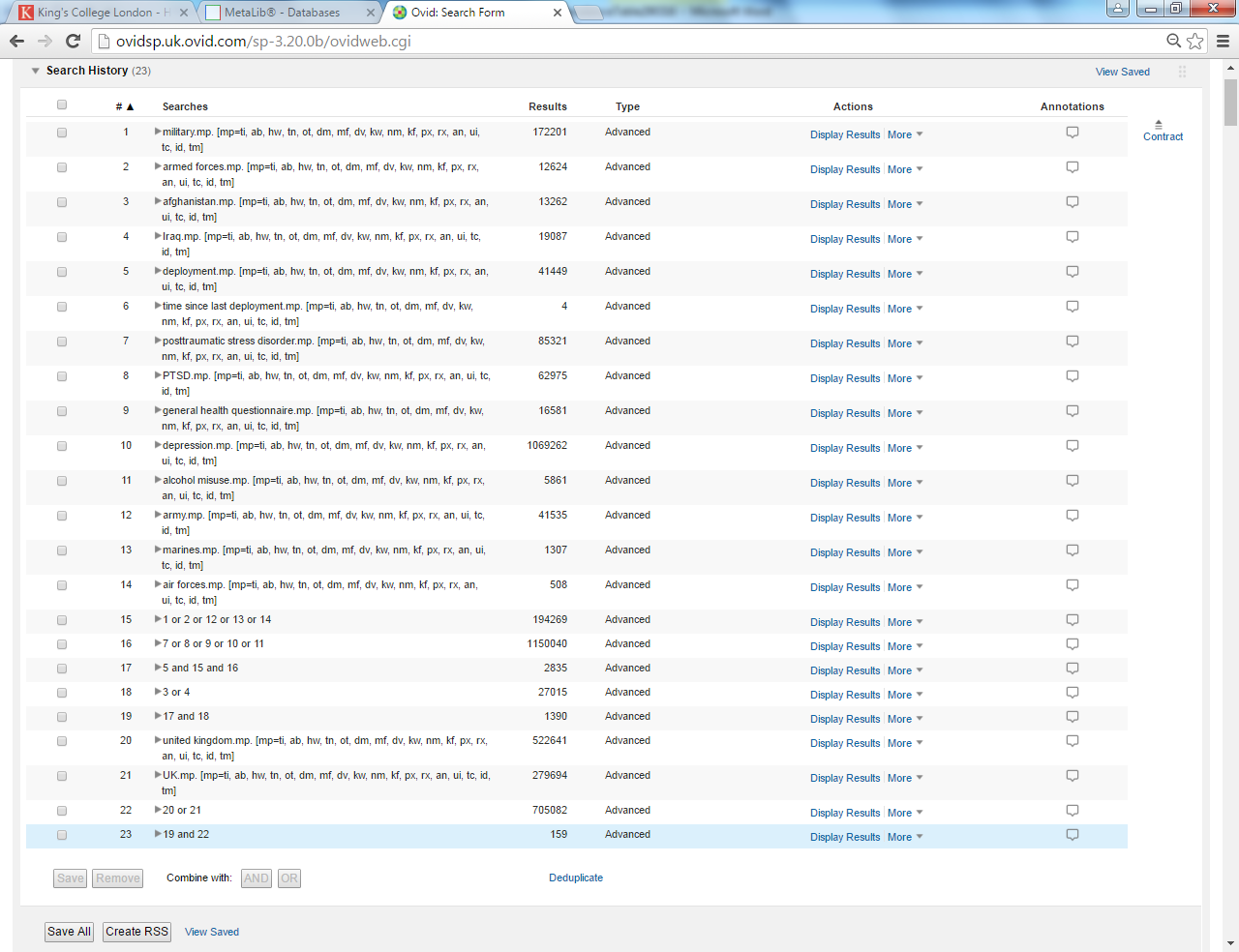

Supplement: Additional file 1: — Search strategy of UK studies that could be potentially relevant for inclusion in this study. (DOCX 195 kb) [file 12888_2016_1038_MOESM1_ESM.docx]
